# The cost of improving nutritional outcomes through food‐assisted maternal and child health and nutrition programmes in Burundi and Guatemala

**DOI:** 10.1111/mcn.12863

**Published:** 2019-08-05

**Authors:** Jessica Heckert, Jef L. Leroy, Deanna K. Olney, Susan Richter, Elyse Iruhiriye, Marie T. Ruel

**Affiliations:** ^1^ Poverty, Health, and Nutrition Division International Food Policy Research Institute Washington, DC; ^2^ Michigan State University, work conducted while affiliated with Poverty, Health, and Nutrition Division International Food Policy Research Institute Washington, DC; ^3^ Department of Health Promotion, Education, and Behavior, Arnold School of Public Health, University of South Carolina, work conducted while affiliated with Poverty, Health, and Nutrition Division International Food Policy Research Institute Washington, DC

**Keywords:** Burundi, cost‐effectiveness, food aid, Guatemala, maternal and child health and nutrition programmes, multisectoral nutrition programmes

## Abstract

Evidence on the cost‐effectiveness of multisectoral maternal and child health and nutrition programmes is scarce. We conducted a prospective costing study of two food‐assisted maternal and child health and nutrition programmes targeted to pregnant women and children during the first 1,000 days (pregnancy to 2 years). Each was paired with a cluster‐randomized controlled trial to evaluate impact and compare the optimal quantity and composition of food rations (Guatemala, five treatment arms) and their optimal timing and duration (Burundi, three treatment arms). We calculated the total and per beneficiary cost, conducted cost consequence analyses, and estimated the cost savings from extending the programme for 2 years. In Guatemala, the programme model with the lowest cost per percentage point reduction in stunting provided the full‐size family ration with an individual ration of corn–soy blend or micronutrient powder. Reducing family ration size lowered costs but failed to reduce stunting. In Burundi, providing food assistance for the full 1,000 days led to the lowest cost per percentage point reduction in stunting. Reducing the duration of ration eligibility reduced per beneficiary costs but was less effective. A 2‐year extension could have saved 11% per beneficiary in Guatemala and 18% in Burundi. We found that investments in multisectoral nutrition programmes do not scale linearly. Programmes providing smaller rations or rations for shorter durations, although less expensive per beneficiary, may not provide the necessary dose to improve (biological) outcomes. Lastly, delivering effective programmes for longer periods can generate cost savings by dispersing start‐up costs and lengthening peak operating capacity.

**Key messages**

- Delivering larger rations (Guatemala) and rations for the full first 1,000 days (pregnancy to age 2 years; Burundi) resulted in the lowest cost per percentage point reduction in stunting.
- Smaller ration sizes and shorter periods of ration eligibility reduced per beneficiary costs, but these less expensive programme models were either not effective or less effective than more expensive ones.
- Extending the programmes for 2 years would have saved between 11% and 18% per beneficiary. Implementing effective programmes for longer periods of time will reduce per beneficiary programme costs.

## 1 INTRODUCTION

Maternal and child undernutrition are global health problems with far‐reaching consequences that are increasingly addressed through multisectoral nutrition programmes aimed at improving the immediate and underlying causes of undernutrition (Black et al., 2013; Ruel & Alderman, 2013). Evidence on programmatic and policy solutions for reducing undernutrition continues to accumulate. Yet evidence on the cost‐effectiveness of nutrition interventions remains limited and is especially scarce for multisectoral nutrition programmes. This limits the ability of donors, implementers, and recipient countries to efficiently allocate limited funds. A typical trade‐off faced by decision makers is between serving more beneficiaries with a less intensive programme or fewer beneficiaries with a more comprehensive programme. Yet there is currently little evidence to guide such decisions (Schieber, Gottret, Fleisher, & Leive, 2007).

Most previous cost studies of nutrition programmes have been of nutrition‐specific programmes that were focussed on single outcome (e.g., iron status; Bhutta et al., 2013; Horton, Shekar, McDonald, Mahal, & Brooks, 2010). Unlike these programmes, multisectoral nutrition programmes typically incorporate inputs from across sectors and aim to improve a range of nutrition and nutrition‐related outcomes for multiple beneficiaries within the household (e.g., mother and child). This complexity makes assessing cost‐effectiveness more challenging. Additionally, the goal of targeting multiple outcomes and the fact that the impacts across these outcomes cannot be easily expressed using a common metric make calculating cost‐effectiveness or cost–benefit ratios challenging.

Herein, we report on the costing results of two large‐scale food‐assisted maternal and child health and nutrition (FA‐MCHN) interventions implemented in Guatemala and Burundi. FA‐MCHN interventions are popular multisectoral nutrition interventions (Bonnard, Haggerty, Swindale, & Bergeron, 2002) and a programme of choice for the United States Agency for International Development's Office of Food for Peace in food insecure environments with a high prevalence of undernutrition (Food and Nutrition Technical Assistance II Project, 2010). Yet there is little evidence on their cost or cost‐effectiveness (Lentz & Barrett, 2013). This costing study was nested in two rigorous programme evaluations that were designed to assess the optimal quantity and composition of the food rations (Guatemala) and the optimal timing and duration of food rations (Burundi) for reducing stunting and its determinants.

The first objective of this study was to determine the cost per beneficiary of the different treatment arm‐based programme models (i.e., the five treatment arms that differed in the quantity and composition of the food rations [Guatemala] and three that differed in the timing and duration of food assistance [Burundi]). For each programme model, we calculated costs if it had been delivered at programme scale (i.e., if it had been delivered to all beneficiaries served by the programme). Second, for each of the different treatment arm‐based programme models, we estimated the cost per beneficiary per percentage point reduction in stunting and conducted a cost consequence analysis for a wide range of programme outcomes. Finally, because lengthy start‐up and closeout periods were required to successfully deliver these programmes, we determined the extent to which these programmes would have operated at a lower cost per beneficiary if they had been implemented for a longer period and thus been able to disperse start‐up costs over more beneficiaries and operate at peak capacity for longer.

## 2 METHODS

### 2.1 The Prevention of Malnutrition in Children under Two Years of Age Program

Detailed descriptions of the Guatemala and Burundi programmes have been published elsewhere (Heckert, Leroy, Bliznashka, Olney, & Richter, 2018; Leroy, Olney, & Ruel, 2016; Leroy, Olney, & Ruel, 2018; Leroy, Sununtnasuk, Heckert, & Olney, 2017). The two programmes, which aimed to prevent undernutrition during the first 1,000 days, included three core components: (a) food assistance, composed of a family and an individual food ration targeted to the mother from pregnancy until the child was 6 months old and then to the child from 6 to 24 months; (b) a health, hygiene, and nutrition behaviour change communication (BCC) strategy that included regular small‐group lessons and other activities; and (c) activities to strengthen the local health care system and promote its use.

In Guatemala, the Maternal and Child Food Diversification Community Program (Programa Comunitario Materno Infantil de Diversificación Alimentaria; PROCOMIDA) was implemented in the Alta Verapaz Department (north central Guatemala) by Mercy Corps. The programme served nearly 53,000 mother–child pairs over the course of implementation. The standard food assistance package, which was a full‐sized family ration (FFR) of rice, beans, and oil paired with an individual ration of corn–soy blend (CSB), was delivered in the catchment area of 95 community health centres. One hundred twenty additional community health centre catchment areas were randomly assigned to one of six study arms for the impact evaluation (five treatment, one control). The treatment arms varied by the size of the family ration (full [FFR], reduced [RFR], or none [NFR]) and the type of individual ration (CSB, lipid‐based nutrient supplement [LNS], or micronutrient powder [MNP]) as follows: FFR + CSB, RFR + CSB, NFR + CSB, FFR + LNS, and FFR + MNP (Table 1). In addition to the food rations, all programme beneficiaries received monthly BCC sessions and recipe demonstrations delivered by trained programme staff, and health service strengthening activities were implemented in the health centres in the five treatment arms.

**Table 1:** Program components of two cluster‐randomized controlled studies of Preventing Malnutrition in Children under 2 Years of Age Approach programmes

	PROCOMIDA (Guatemala)					*Tubaramure* (Burundi)		
Designed to evaluate	Optimal composition of the individual ration and size of the family ration					Optimal timing and duration of food assistance		
Study arm	FFR + CSB	RFR + CSB	NFR + CSB	FFR + LNS	FFR + MNP	T24	T18	TNFP
Family ration	Full^a^	Reduced	None	Full	Full	Yes^a^	Yes	Yes
Individual ration^b^	CSB^c^	CSB	CSB	LNS^d^	MNP^e^	CSB and oil^f^	CSB and oil	CSB and oil
Start of food ration eligibility	Pregnancy	Pregnancy	Pregnancy	Pregnancy	Pregnancy	Pregnancy	Pregnancy	Birth
End of food ration eligibility	24 months	24 months	24 months	24 months	24 months	24 months	18 months	24 months
BCC	Yes	Yes	Yes	Yes	Yes	Yes	Yes	Yes
Health service strengthening	Yes	Yes	Yes	Yes	Yes	Yes	Yes	Yes

Abbreviations: BCC, behaviour change communication; CSB, corn–soy blend; FFR, full family ration; LNS, lipid‐based nutrient supplement; MNP, micronutrient powder; NFR, no family ration; PROCOMIDA, Programa Comunitario Materno Infantil de Diversificación Alimentaria; RFR, reduced family ration.

a In Guatemala, the full monthly ration was 6 kg of rice, 4 kg of beans, and 1,850 g of fortified vegetable oil, and the reduced was 3 kg of rice, 3 kg of beans, and 925 g of fortified vegetable oil. In Burundi, the monthly family ration was 12 kg of CSB and 1,200 g of fortified vegetable oil.

b Provided to women who were pregnant or had a child between 0 and 5 months old and children 6–23 months.

c Women and children received 4 kg each month.

d Women received 30 sachets (20 g), and children received 60 sachets (10 g) each month.

e Women and children received 60 sachets (2 g) each month.

f Women received 6 kg of CSB and 600 g of fortified vegetable oil, and children received 3 kg of CSB and 300 g of fortified vegetable oil each month.

*Tubaramure* (a Kirundi word meaning “let's help them grow”) was implemented in the provinces of Cankuzo and Ruyigi in eastern Burundi by a Catholic Relief Services–led consortium, which also included CARITAS Burundi, Food for the Hungry, and International Medical Corps. Approximately 36,000 mother–child pairs were enrolled in the programme. The standard programme provided food rations (family and individual rations of CSB and oil) from pregnancy until the child was 24 months old and was implemented in 205 *collines* (smallest administrative division in Burundi). An additional sixty *collines* were randomly assigned to one of four study arms (three treatment, one control). The T24 arm received the standard programme of food rations from pregnancy until the child was 24 months old (Table 1). Beneficiaries in the T18 arm received food rations until the child was 18 months and all other benefits until the child was 24 months. Beneficiaries in the TNFP (no food ration during pregnancy) arm received all benefits of the standard programme, except for the food rations during pregnancy. Twice each month in all treatment arms and the standard programme, beneficiary mothers received a BCC session delivered by volunteer leader mothers who were trained monthly by programme staff. All community health centres in the two provinces benefited from health service strengthening activities.

### 2.2 Estimating costs

We first describe how we calculated the cost of programme activities for the entire programme as implemented (standard programme, plus treatment arms) in each country, the cost of delivering each of the treatment arm–based program models if it were implemented at scale, and the cost per beneficiary (including food). We then explain our approach to assessing “cost‐effectiveness” in the context of a programme aimed at improving a range of outcomes. Finally, we describe how we calculated the cost per beneficiary for each programme model under a hypothetical 2‐year extension of the programme.

#### 2.2.1 Cost of programme activities

We used a prospective costing design and calculated costs using the activity‐based costing ingredients method (ABC‐I; Kaplan & Anderson, 2004; Tan‐Torres Edejer et al., 2003). The first step was to identify activity‐based cost centres (AB‐CCs), which aggregate costs based on programme activities. The AB‐CCs were defined so that they were mutually exclusive and exhaustive of all programme activities. A description of programme activities and staff responsibilities was developed based on the programme proposals developed by the implementing non‐governmental organizations. For each programme, key programme staff participated in an initial workshop to further elaborate the programme description, and annual interviews thereafter were used to update it. The programme descriptions included nine AB‐CCs for PROCOMIDA and eight for *Tubaramure* (listed in Table 2 and described in Table S1). The first four were related to the delivery of the core programme components. The remaining AB‐CCs were the activities to support, monitor, and manage the core activities. Activities for each year were classified as either start‐up (those conducted during the development of the programme that would not need to be repeated to sustain the programme, such as the design of the BCC lessons) or post‐start‐up activities.

**Table 2:** Programme activity costs and start‐up costs by activity‐based costing centre

	PROCOMIDA, Guatemala		*Tubaramure*, Burundi	
Activity‐based costing centre	Cost (2015 USD)	% of total programme costs	Cost (2015 USD)	% of total programme costs
1. Supply and logistics of food commodity and supplement distribution				
Start‐up	25,745	0.1	92,211	0.4
Total	3,023,382	10.9	3,218,377	13.6
2. Food ration and supplement distribution				
Start‐up	934,602	3.4	88,028	0.4
Total	5,296,188	19.1	7,184,147	30.4
3. BCC development and execution				
Start‐up	1,288,931	4.7	1,055,722	4.5
Total	4,722,974	17.1	3,058,398	12.9
4. Institutional strengthening of health services				
Start‐up	22,202	0.1	123,970	0.5
Total	3,268,831	11.8	2,009,724	8.5
5. Monitoring and evaluation				
Start‐up	947,101	3.4	180,722	0.8
Total	3,004,106	10.8	1,146,683	4.9
6. Training and supervision of programme staff				
Start‐up	—	—	226,578	1.0
Total	1,495,287	5.4	1,985,693	8.4
7. Advocacy, promotion, and social mobilization				
Start‐up	300,613	1.1	112,460	0.5
Total	1,052,695	3.8	591,950	2.5
8. Management, planning, and administration				
Start‐up	155,436	0.6	511,135	2.2
Total	4,407,653	15.9	4,430,654	18.8
9. Systematic information management				
Start‐up	1,127,282	4.1		
Total	1,426,932	5.2		
Total start‐up	4,780,912	17.3	2,390,826	10.1
Total costs	27,698,046		23,625,626	

*Note*. Costs in this table only include the cost of programme activities and do not include the cost of food rations and supplements.

Abbreviations: BCC, behaviour change communication; PROCOMIDA, Programa Comunitario Materno Infantil de Diversificación Alimentaria.

The second step was to collect data on the type (e.g., labour and materials) and quantity of inputs required for each activity from programme documents, workshops, individual interviews, and observations of programme activities. Annual interviews were conducted with individual members of the programme staff. For field activities (e.g., food distribution and delivery of BCC lessons), data on the allocation of labour and materials were collected by direct observation at randomly selected field sites. The implementing non‐governmental organizations provided detailed finance information, which was used to determine the cost of each input.

The quantity of inputs required for each activity and their unit costs were entered into spreadsheets. Inputs were classified as either a capital cost (one‐time expenses, such as equipment) or recurrent cost (for inputs such as labour and materials). Capital costs were annualized by allocating them across the remaining programme years using a discount rate of 3%. Recurrent costs were directly allocated to the year that they were incurred. We calculated the cost of each activity in each year according the amount of each input required and the cost of the input. To account for indirect costs, we added 15% to the cost of each activity. Because the costing literature provides no clear guidance on which indirect rate to use, we used 15% as an estimated average of allowable indirect costs across programme funders and implementers. Importantly, the choice of the indirect cost rate does not affect the primary results of our study (i.e., the difference in costs between programme models).

The costs of start‐up and post‐start‐up activities were handled separately. The cost of each start‐up activity was annualized across the remaining programme years and converted to the base year of 2009 by adjusting for annual inflation using a GDP deflator of 3% (Dhaliwal, Duflo, Glennerster, & Tulloch, 2013; Tan‐Torres Edejer et al., 2003). Then, the present value of the annualized start‐up activity cost was calculated by dividing the annual discounted cost by the annualization factor and converting it to the year of analysis (2015; Dhaliwal et al., 2013). These values were then equally divided among the years the activity was implemented and each subsequent year. The start‐up activity costs were summed for each AB‐CC to calculate the annual AB‐CC‐specific start‐up activity costs. For post‐start‐up activities, costs were adjusted for the annual rate of inflation using the same GDP deflator. The present value of the annual costs was then calculated, and costs were inflated to the year of analysis (2015). The annual costs of all post‐start‐up activities within an AB‐CC were summed to obtain post‐start‐up activity costs for each AB‐CC for each programme year (Phillips & Fiedler, 2007). The total cost of each AB‐CC was the sum of its start‐up and post‐start‐up costs. The total cost of programme activities was the sum of the cost of all AB‐CCs. All costs are reported in 2015 U.S. dollars (USD).

#### 2.2.2 Cost of activities for each treatment arm–based programme model

We calculated the hypothetical programme activity cost if each treatment arm was implemented at programme scale (i.e., if all programme beneficiaries received that treatment). The detailed programme description was used to identify which activities would have been conducted differently, to adjust the quantity of the inputs needed to implement these activities, and to estimate the cost of programme activities for each treatment arm–based programme models (Puett et al., 2013). Because the programmes had target beneficiary numbers and monitored enrolment to reach these goals, we assumed for those treatment arms with lower programme uptake that the corresponding treatment arm–based programme model would have expanded services to reach the target number of beneficiaries and adjust inputs accordingly.

#### 2.2.3 Cost of programme activities per beneficiary

The total cost of programme activities for each treatment arm–based programme model was divided by the total number of beneficiary‐months to produce the monthly cost per beneficiary. To calculate the total number of beneficiary‐months, we used data collected by each programme on the number of beneficiaries served each month (Figure S1). The per beneficiary cost was then multiplied by the average months of programme participation to calculate the total cost of programme activities per beneficiary.

#### 2.2.4 Cost of food rations and supplements per beneficiary

To accurately calculate the per beneficiary cost of food rations and supplements, which varied in quantity by treatment arm and by who was receiving the individual ration (the mother or the child), the cost of the food rations and supplements was calculated separately from the programme activity costs. The cost of food commodities (including shipping) was obtained from the United States Agency for International Development commodity price estimates (United States Agency for International Development, 2016). The cost of LNS and MNP (including shipping) was obtained from invoices. The monthly cost of the household and individual rations and supplements (calculated separately for whether the primary beneficiary was the woman or the child) was multiplied by the average number of months that the ration was received and summed to determine the cost per beneficiary for the duration of the programme.

#### 2.2.5 Total cost per beneficiary

The per beneficiary costs of programme activities, food rations, and supplements were summed to calculate the total cost per beneficiary.

### 2.3 Comparing programme costs and impacts

For estimates of the impact of PROCOMIDA and *Tubaramure*, we drew on results from the cluster‐randomized controlled programme evaluations. We first calculated the cost per beneficiary per percentage point reduction in stunting. Then, to address the shortcomings of using a single cost‐effectiveness ratio for a multisectoral nutrition programme with impacts across multiple outcomes at the child, mother, and household level, we used a cost‐consequence approach to compare the full scope of programme impacts (Mauskopf, Paul, Grant, & Stergachis, 1998). To do so, we drew on the programme impact pathways that were identified at the outset of the programmes (Olney et al., 2013a; Olney et al., 2013b) and compared them across the study arms.

### 2.4 Effect of increasing programme duration on cost per beneficiary

To determine the extent to which programme duration affected the cost per beneficiary, we estimated the hypothetical programme costs if the Guatemala and Burundi programmes had operated for two additional years at peak capacity. Two years represent a meaningful increase in programme length (around 33%), while not being so long that programmes would need to replace large capital goods (e.g., vehicles) or face new start‐up costs (e.g., to refresh the BCC strategy). We assumed that during the two additional years, the programme would have implemented the same post‐start‐up activities at the same cost as during the peak enrolment year (2012). For each of the two extension years, we used the costs of post‐start‐up activities in 2012 and redistributed start‐up costs to include the two additional years. The costs of all pre‐2012 activities (including the two extension years) were adjusted using a revised annualization factor, inflation rate, and deflation rate. The cost of post‐start‐up activities incurred after 2012 was not altered, as the parameters for these years would have remained the same.

To inform the cost‐per‐beneficiary calculation for the hypothetical 2‐year extension, we set the number of beneficiaries being served each month during the two additional years to the average monthly number of beneficiaries during the peak enrolment months of late 2011 to early 2012 for *Tubaramure* and 2013 for PROCOMIDA. Using this information and assuming the same programme impact, we recalculated the cost per beneficiary and cost per beneficiary per percentage point reduction in stunting.

## 3 RESULTS

### 3.1 Cost of PROCOMIDA in Guatemala

#### 3.1.1 Programme activity costs

**Figure 1:**
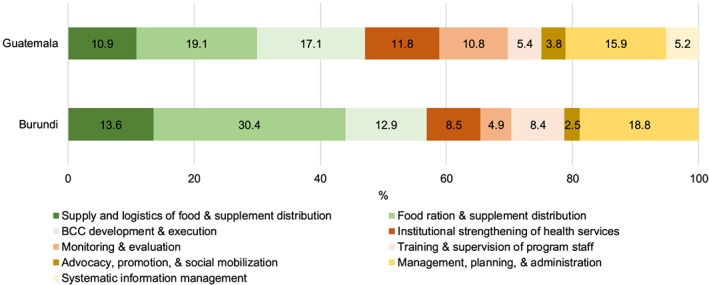
Distribution of programme activity costs by activity‐based costing centre. BCC = behaviour change communication

The total cost of PROCOMIDA activities, which included the standard programme in the nonstudy area and all five treatment arms in the study area but did not include the cost of rations and supplements, was 27.7 million USD (Table 2). Distributing the food rations and supplements was the most expensive AB‐CC (19.1% of programme activity costs); when combined with the supply and logistics of food commodity and supplement distribution, the delivery of food rations accounted for 30.0% of total programme activity costs (Figure 1). The second most costly AB‐CC was BCC development and execution (17.1%), which required extensive staff time to deliver and employed the largest number of staff members. Management, planning, and administration, which required the staff time from managers and headquarters staff with higher salaries, ranked third in the percentage of total activity cost.

Start‐up costs accounted for 17.3% of total programme activity costs; the four AB‐CCs with the largest start‐up costs were food ration and supplement distribution, development and execution of the BCC strategy, monitoring and evaluation, and systematic information management (Table 2). The other AB‐CCs either incurred no or minimal start‐up costs.

#### 3.1.2 Programme activity costs by treatment arm–based programme model

The least expensive programme model was RFR + CSB at an estimated 26.1 million USD total and 721 USD per beneficiary, because the programme stored and transported smaller quantities of food (Table 3). The NFR + CSB programme model was the most expensive at 28.4 million USD total and 785 USD per beneficiary; the increased expenses are attributable to lower programme participation, which would have required the programme to expand geographically to reach the same number of beneficiaries (Heckert, Leroy, Bliznashka, Olney, & Richter, 2018). The total cost of programme activities for any of the three programme models that provided the FFR along with CSB, LNS, or MNP was approximately 27.5 million USD total and 760 USD per beneficiary. Total start‐up costs were between 15.0% and 16.2% of programme activity costs and highest in the programme models delivering LNS and MNP.

**Table 3:** Costs by programme model when implemented at scale, PROCOMIDA

	FFR + CSB	RFR + CSB	NFR + CSB	FFR + LNS	FFR + MNP
Total and start‐up costs by AB‐CC (USD)^a^					
1. Supply and logistics of food commodity distribution	2,980,894	2,479,509	1,950,462	2,458,519	2,455,669
Start‐up costs	25,579	25,579	24,004	25,579	25,579
2. Food ration and supplement distribution	5,196,771	4,329,329	4,698,884	5,568,695	5,480,954
Start‐up costs	624,489	622,310	638,149	1,105,739	1,105,739
3. BCC development and execution	4,919,852	4,919,852	5,918,225	4,980,536	4,980,536
Start‐up costs	1,194,556	1,194,556	1,210,368	1,255,240	1,255,240
4. Institutional strengthening of health services	3,268,831	3,268,831	3,985,049	3,268,831	3,268,831
Start‐up costs	22,202	22,202	22,202	22,202	22,202
5. Monitoring and evaluation	2,801,525	2,801,525	3,107,456	2,846,976	2,846,559
Start‐up costs	821,158	821,158	847,363	821,158	821,158
6. Training and supervision of programme staff	1,495,287	1,495,287	1,990,807	1,495,287	1,495,287
Start‐up costs	—	—	—	—	—
7. Advocacy, promotion, and social mobilization	1,020,524	1,020,524	1,020,524	1,052,632	1,052,632
Start‐up costs	290,695	290,695	290,695	300,550	300,550
8. Management, planning, and administration	4,406,904	4,406,904	4,419,857	4,406,904	4,406,904
Start‐up costs	154,687	154,687	154,687	154,687	154,687
9. Systematic information management	1,390,516	1,390,516	1,338,997	1,522,026	1,522,026
Start‐up costs	1,095,576	1,095,576	1,076,156	1,167,070	1,167,070
Total cost of programme activities	27,481,104	26,112,277	28,430,261	27,600,406	27,509,398
Total start‐up costs	4,228,942	4,226,763	4,263,624	4,852,225	4,852,225
Monthly cost of programme activities per beneficiary^b^					
Total number of beneficiary‐months^b^	1,050,166	1,050,166	1,050,166	1,050,166	1,050,166
Monthly cost of programme activities per beneficiary^b^ (USD)^a^	26.17	24.86	27.07	26.28	26.20
Monthly cost of ration and supplement per beneficiary^b^ (USD)^a^					
Family ration	8.68	5.05	—	8.68	8.68
Individual ration or supplement for pregnant woman or mother of a child <6 months old	2.38	2.38	2.38	2.64	2.67
Individual ration or supplement for children 6–23 months	2.38	2.38	2.38	2.64	2.11
Months that each programme component was received (no.)					
Ration for pregnant woman or mother of child <6 months old^c^	11	11	11	11	11
Ration for child aged 6–23 months	18	18	18	18	18
Family ration	29	29	29	29	29
Duration of programme participation (including BCC and health services)	29	29	29	29	29
Total costs per beneficiary^b^ (USD)^a^					
Programme activities for the duration of the programme	758.88	721.08	785.09	762.18	759.66
Food rations and supplements for the duration of the programme	320.82	215.57	69.05	328.23	319.14
Combined cost of food rations, supplements, and programme activities	1,079.91	936.65	854.14	1,090.41	1,078.18

Abbreviations: AB‐CC, activity‐based cost centre; BCC, behaviour change communication; CSB, corn–soy blend; FFR, full family ration; LNS, lipid‐based nutrient supplement; MNP, micronutrient powder; NFR, no family ration; PROCOMIDA, Programa Comunitario Materno Infantil de Diversificación Alimentaria; RFR, reduced family ration.

a 2015 USD.

b Beneficiary refers to a mother–child pair.

c Women, on average, enrolled in PROCOMIDA in time to receive five monthly rations during pregnancy.

Underlying the differences in programme activity costs were differences in the costs of seven of the nine AB‐CCs (Table 3). The costs related to the distribution of the food rations and supplements (AB‐CCs 1 and 2) varied by the amount and type of food rations and supplements, with programme model FFR + CSB being the most expensive. BCC development and execution, institutional strengthening of health services, monitoring and evaluation, and training and supervision of programme staff were all more expensive in the NFR + CSB programme model due to the need to cover a larger geographic area. Systematic information management was more expensive for FFR + LNS and FFR + MNP to accommodate the additional monitoring required for LNS and MNP.

#### 3.1.3 Cost of food rations and supplements per beneficiary

The total monthly cost of the FFR was 8.68 USD compared with 5.05 USD for the RFR. The monthly costs of the MNP supplement were 2.11 USD for children 6–23 months and 2.67 USD for mothers. The monthly costs of CSB and LNS were 2.38 and 2.64 USD, respectively (neither varied by whether the mother or child was the recipient).

The largest differences in the total cost of the family and individual rations over the course of the programme were driven by the size of the family ration, which was smaller in the RFR + CSB and non‐existent in the NFR + CSB programme models; the cost of the combined family and individual rations for these programme models were 69.05 and 215.57 USD, respectively. The total cost of food rations and supplements was highest in the programme models that included the FFR with either CSB, LNS, or MNP (320.82, 328.23, and 319.14 USD, respectively).

#### 3.1.4 Total cost per beneficiary

The total combined cost of the family and individual food rations and programme activities was similar in the FFR + CSB, FFR + LNS, and FFR + MNP programme models and ranged from 1,078 USD to 1,090 USD per beneficiary. The RFR + CSB model costs 937 USD per beneficiary. Despite needing to expand the geographic scope to reach the target number of beneficiaries, omitting the family ration meant that the NFR + CSB model would have been the least expensive at 854 USD per beneficiary.

### 3.2 Comparing costs and impacts of PROCOMIDA

PROCOMIDA reduced stunting by 11.1 and 6.5 percentage points in FFR + CSB and FFR + MNP, respectively, at 24 months of age (Table 4). No other PROCOMIDA arm reduced the prevalence of stunting at 24 months relative to the control group. Due to the similar costs of these two programme models, FFR + CSB had a lower cost per beneficiary per percentage point reduction in stunting (96 USD) than FFR + MNP (166 USD).

**Table 4:** Costs and impacts by programme model, PROCOMIDA

Programme impacts	FFR + CSB	RFR + CSB	NFR + CSB	FFR + LNS	FFR + MNP
Linear growth					
Cost per beneficiary (USD)^a^	1,079.91	936.65	854.14	1,090.41	1,078.18
Impact on stunting (pp)^b^	−11.1 ± 4.0*	—	—	—	−6.5 ± 3.6*
Cost per beneficiary per pp reduction in stunting (USD)^a^	97.29	—	—	—	165.87
Other programme impacts^c^					
Child					
Anaemia (24 months)	X	—	—	—	—
Motor milestones (24 months)	—	—	—	—	—
Language milestones (24 months)	—	—	—	—	—
Breastfeeding initiated immediately after birth	✓	✓	—	—	✓
Exclusively breastfed (4 months)	✓	✓	✓	✓	✓
Bottle‐fed in past 24 hr (6 months)	✓	✓	✓	✓	✓
Minimum meal frequency (24 months)	✓	✓	—	✓	✓
Minimum dietary diversity (24 months)	✓	—	—	—	✓
Minimal acceptable diet (24 months)	✓	—	—	—	✓
Child clean (24 months)	✓	—	—	—	✓
Illness past 2 weeks (24 months)	—	—	—	—	—
Received treatment for fever (24 months)	✓	✓	—	✓	—
Length recorded on health card (24 months)	✓	✓	—	✓	✓
Mother					
Anaemia (24 months)	X	X	—	—	—
Bodyweight (24 months)	X	X	X	—	—
Dietary diversity (24 months)	—	—	—	—	—
Had at least four prenatal visits	—	—	—	—	—
Knowledge					
Pregnancy danger signs (1 month)	✓	✓	✓	—	✓
To breastfeed immediately after birth (1 month)	✓	✓	—	—	—
To use a cup, not a bottle (6 months)	✓	✓	✓	✓	✓
To introduce foods at 6 months (6 months)	—	—	—	—	—
Number of five key handwashing times (24 months)	✓	✓	✓	✓	✓
Household					
Treated drinking water (24 months)	—	—	—	✓	✓
Exterior clean (24 months)	✓	—	—	—	—
Interior clean (24 months)	—	—	—	—	✓
Household hunger (12 months)	✓	—	—	—	✓

*Note*. Data were collected at pregnancy and when the child was 1, 4, 6, 9, 12, 18, and 24 months. Individual indicators are only shown for the time point when they are most relevant. The time referenced refers to the age of the child at the survey. ✓ = Significant improvement in the outcome relative to the control group. X = Significant worsening in the outcome relative to the control group. — = No significant effect.

Abbreviations: CSB, corn–soy blend; FFR, full family ration; LNS, lipid‐based nutrient supplement; MNP, micronutrient powder; NFR, no family ration; PROCOMIDA, Programa Comunitario Materno Infantil de Diversificación Alimentaria; RFR, reduced family ration.

a 2015 USD.

b Impact estimates from Olney, Leroy, Bliznashka, and Ruel (2018).

c Impacts from Heckert et al. (2018).

* *p* < .05 (programme impact was significant).

In addition to reducing stunting, PROCOMIDA improved other outcomes along the programme impact pathways. It increased adoption of optimal infant and young child feeding and childcare practices, improved maternal health and nutrition knowledge, and in some treatment arms improved household cleanliness and reduced household hunger. In addition to these positive programme effects, however, PROCOMIDA increased maternal and child anaemia in the FFR + CSB arm and led to increased maternal bodyweight at 24 months post‐partum in all three CSB arms. These potentially negative effects were not found in the arms that provided LNS or MNP as the individual ration.

### 3.3 Cost of *Tubaramure* in Burundi

#### 3.3.1 Programme activity costs

The total cost of *Tubaramure* programme activities (not including food rations) was 23.6 million USD. Food ration distribution accounted for the largest share (30.4%) of programme activity costs (Table 2). When combined with the supply and logistics of food commodity distribution, the delivery of food rations made up 44.0% of total programme activity costs. The next most costly activities were management, planning, and administration (18.8% of total activities) and the development and execution of the BCC strategy (12.9%). Start‐up costs constituted 10.1% of programme activities costs. The development and execution of the BCC strategy and the management, planning, and administration activities incurred the largest share of start‐up costs.

#### 3.3.2 Programme activity costs by treatment arm–based programme model

The T24 treatment arm–based programme model was the most expensive at 23.9 million USD total (or 453 USD per beneficiary) if implemented at scale (Table 5). The TNFP model was only slightly less expensive at 23.7 million USD total and 449 USD per beneficiary. The T18 model was 22.6 million USD total and 427 USD per beneficiary. At around 10% of total activity costs, start‐up costs were similar for all three. The largest differences in costs across treatment arm–based programme models were attributable to the food distribution activities; T18 and TNFP stored and transported smaller amounts of food, and food distribution activities ended 6 months earlier in T18. The differences among programme models for the costs of monitoring and evaluation and the training and supervision of programme staff were relatively small, and in both cases, T18 would have been the least expensive, and T24 would have been the most expensive. The remaining AB‐CCs did not differ across programme models.

**Table 5:** Costs per programme model when implemented at scale, *Tubaramure*

	T24	T18	TNFP
Total and start‐up costs by AB‐CC (USD)^a^			
1. Supply and logistics of food commodity distribution	3,375,300	2,957,281	3,259,431
Start‐up costs	92,211	92,211	92,211
2. Food ration and supplement distribution	7,208,414	6,479,480	7,195,249
Start‐up costs	88,028	88,028	88,028
3. BCC development and execution	3,058,398	3,058,398	3,058,398
Start‐up costs	1,055,722	1,055,722	1,055,722
4. Institutional strengthening of health services	2,009,724	2,009,724	2,009,724
Start‐up costs	123,970	123,970	123,970
5. Monitoring and evaluation	1,183,499	1,146,582	1,181,655
Start‐up costs	180,722	180,722	180,722
6. Training and supervision of programme staff	2,011,957	1,911,733	2,011,075
Start‐up costs	226,578	226,578	226,578
7. Advocacy, promotion, and social mobilization	590,492	590,492	590,492
Start‐up costs	111,001	111,001	111,001
8. Management, planning, and administration	4,430,654	4,430,654	4,430,654
Start‐up costs	511,135	511,135	511,135
Total cost of programme activities	23,868,438	22,584,344	23,736,677
Total start‐up costs	2,389,367	2,389,367	2,389,367
Monthly cost of programme activities per beneficiary^b^			
Total number of beneficiary‐months^b^	1,427,134	1,427,134	1,427,134
Monthly cost of programme activities per beneficiary^b^ (USD)^a^	16.72	15.82	16.63
Monthly cost of ration and supplement per beneficiary^b^ (USD)^a^			
Pregnant women or mothers of children <6 months old	4.36	4.36	4.36
Child aged 6–23 months	2.18	2.18	2.18
Family	8.72	8.72	8.72
Months that each programme component was received (no.)			
Ration for pregnant women or mothers of children <6 months old^c^	9	9	5
Ration for child aged 6–23 months	18	12	18
Family ration	27	21	23
Duration of programme participation (including BCC and health services)	27	27	27
Total cost per beneficiary^b^ (USD)^a^			
Programme activities for the duration of the programme	451.57	427.27	449.07
Food rations for the duration of the programme	314.06	248.63	261.72
Combined cost of programme activities and food rations	765.63	675.91	710.79

Abbreviation: AB‐CC, activity‐based cost centre.

a 2015 USD.

b Beneficiary refers to a mother–child pair.

c Women in T24 and T18, on average, enrolled in time to receive three monthly rations during pregnancy. Women in TNFP received, on average, five monthly rations during the time their child was <6 months old.

#### 3.3.3 Cost of food rations

The monthly cost of the combined individual and family rations for households with a pregnant mother or a child younger than 6 months was 13.08 USD. For a household with a child between 6 and 23 months, the cost was 10.90 USD per month. The total cost of food rations per beneficiary (i.e., for the duration of programme participation) was highest for T24 (314 USD), followed by TNFP (262 USD), and T18 (249 USD).

#### 3.3.4 Total costs per beneficiary

The total combined cost of programme activities and food rations per beneficiary was highest in T24 (766 USD) and slightly less expensive in TNFP (711 USD) and T18 (676 USD).

### 3.4 Comparing costs and impacts of *Tubaramure*

*Tubaramure* had a significant impact on stunting in the T24 (−7.4 percentage points) and T18 (−5.7 percentage points) arms and a marginally significant impact in TNFP (−4.6 percentage points; Olney et al., 2018). The cost per beneficiary per percentage point reduction in stunting was estimated at 103, 119, and 155 USD, respectively (Table 6). In addition to reducing stunting, *Tubaramure* had positive impacts on a range of child‐, maternal‐, and household‐level outcomes. The programme improved child haemoglobin and development, reduced child morbidity, and improved infant and young child feeding practices. In mothers, the programme was found to reduce anaemia, increase dietary diversity, and improve prenatal care seeking. *Tubaramure* also improved maternal knowledge in several health and nutrition‐related domains, improved household food security and handwashing practices, and decreased the proportion of households not treating their drinking water.

**Table 6:** Costs and impacts by programme model, *Tubaramure*

Program impacts	T24	T18	TNFP
Linear growth			
Cost per beneficiary (USD)^a^	765.63	675.91	710.79
Impact on stunting (pp)^b^	−7.4 ± 3.4*	−5.7 ± 3.4*	−4.6 ± 3.4**
Cost per beneficiary per pp reduction in stunting (USD)^a^	103.46	118.58	154.52
Other programme impacts^c^			
Child (0–23 months)			
Haemoglobin	✓	✓	—
Motor milestones (12–23 months)	—	—	✓
Language milestone (4–23 months)	✓	✓	—
Illness past 2 weeks	✓	✓	✓
Received treatment for fever, pp	✓	—	✓
Consumption of iron‐rich foods (6–23 months)	✓	✓	✓
Minimum meal frequency (6–23 months)	—	✓	✓
Minimum dietary diversity (6–23 months)	✓	✓	✓
Minimal acceptable diet (6–23 months)	—	✓	✓
Length recorded on vaccination card	—	—	✓
All clean	—	✓	✓
Mother			
Anaemia	✓	—	—
Dietary diversity	✓	✓	✓
Total number of prenatal visits	✓	✓	—
Had at least four prenatal visits	✓	✓	✓
Pregnancy month at first prenatal visit	✓	✓	✓
Knowledge			
Number of five key handwashing times	✓	✓	✓
Ash is an appropriate handwashing product	✓	✓	✓
Liquids should not be introduced before 6 months	✓	✓	✓
Foods should not be introduced before 6 months	✓	✓	✓
Correct feeding frequency for child 6–9 months	—	✓	✓
Correct feeding frequency for child 12–23 months	—	✓	—
Yellow/orange fruits and vegetables as source vitamin A	✓	✓	✓
CSB is a source of iron	✓	✓	✓
Sick child (<6 months) should not be fed less breastmilk	✓	✓	✓
Sick child (>6 months) should not be fed less food	✓	✓	✓
Household			
Treated drinking water	—	✓	✓
Severely food insecure	✓	✓	✓
Household hunger	—	—	✓
Household dietary diversity	—	✓	—
Wash hands with soap after defecation	✓	✓	✓
Exterior clean	—	—	—
Interior clean	—	✓	—

*Note*. ✓ = Significant improvement in the outcome relative to the control group; — = No significant effect.

a 2015 USD.

b Impact estimates were taken from Leroy, Olney, and Ruel (2018).

c Impact estimates were taken from Leroy, Heckert, Cunningham, and Olney (2014). Estimates refer to children 0–23 months old unless otherwise indicated.

* *p* < .05 (programme impact was significant).

** *p* < .10 (programme impact was marginally significant).

### 3.5 Changes in the cost per beneficiary when increasing programme duration

Under the 2‐year extension scenario, the total cost per beneficiary of delivering the programme decreased by approximately 15% (from between 721 and 785 USD to between 612 and 683 USD) for PROCOMIDA and by approximately 30% (from between 427 and 452 USD to between 300 and 312 USD) for *Tubaramure* (Table 7). Once combined with the cost of the food rations and supplements (which would not have changed), the total cost of PROCOMIDA per beneficiary was approximately 11% lower for each of the programme models, and the cost per beneficiary per percentage point reduction in stunting was 87 USD for FFR + CSB and 147 USD for FFR + MNP. For *Tubaramure*, after including the cost of food, the cost per beneficiary was 18% lower, and the cost per beneficiary per percentage point reduction in stunting was 86, 96, and 125 USD for T24, T18, and TNFP, respectively.

**Table 7:** Decreasing the cost per beneficiary of PROCOMIDA and *Tubaramure* under a 2‐year extension scenario

	PROCOMIDA, Guatemala					*Tubaramure*, Burundi		
	FFR + CSB	RFR + CSB	NFR + CSB	FFR + LNS	FFR + MNP	T24	T18	TNFP
Total cost of programme activities (USD)^a^	36,076,359	34,181,366	38,155,214	35,547,946	35,541,929	32,027,360	30,779,461	31,913,036
Total number of beneficiary‐months	1,619,257	1,619,257	1,619,257	1,619,257	1,619,257	2,770,704	2,770,704	2,770,704
Monthly cost of programme activities per beneficiary (USD)^a^	22.28	21.11	23.56	21.95	21.95	11.56	11.11	11.52
Total cost of programme activities per beneficiary (USD)^a^	646.11	612.17	683.34	636.64	636.54	312.10	299.94	310.99
Combined cost of programme activities and food rations (USD)^a^	966.93	827.74	752.39	964.87	955.68	626.16	548.57	572.71
Cost per beneficiary per pp reduction in stunting (USD)^a^	87.11	—	—	—	147.03	85.62	96.24	124.50*

*Note*. — = Treatment arm did not have a significant impact on stunting.

Abbreviations: CSB, corn–soy blend; FFR, full family ration; LNS, lipid‐based nutrient supplement; MNP, micronutrient powder; NFR, no family ration; PROCOMIDA, Programa Comunitario Materno Infantil de Diversificación Alimentaria; RFR, reduced family ration.

a 2015 USD.

* *p* < .10 (impact was marginally significant).

## 4 DISCUSSION

This study, carried out in Guatemala and Burundi, is the first prospective cost study of large‐scale FA‐MCHN programmes. Our first objective was to determine the programme cost per beneficiary. In Guatemala, providing households with an FFR with either CSB, LNS, or MNP as the individual ration during pregnancy and up to the child's second birthday (~1,000‐day period), along with BCC and health‐strengthening activities, costs approximately 1,080 USD per beneficiary. Providing a smaller family ration or forgoing it reduced programme costs to 940 and 850 USD per beneficiary, respectively. The price of delivering the full Burundi programme (T24) was approximately 770 USD per beneficiary. Reducing the duration of food assistance to the child by 6 months (stopping at 18 instead of 24 months of age; T18) reduced the cost to approximately 680 USD; and withholding food assistance during pregnancy (TNFP) reduced the cost to 710 USD per beneficiary. Reducing the duration of food assistance did not substantially reduce the cost of programme activities (only the cost of food), as food distributions still required similar logistical and managerial inputs, even though fewer beneficiaries were attending any given distribution. This is consistent with findings that costs of child health days were largely driven by the number of sites, not the number of children treated (Fiedler et al., 2014).

We found no other comprehensive cost studies of large‐scale FA‐MCHN programmes with which to compare these estimates. A multicountry study of food assistance implemented by the World Food Program from 2010 to 2012 estimated monthly per beneficiary food delivery costs of 11.46 USD in Ecuador, 6.41 USD in Uganda, 9.84 USD in Yemen, and 10.27 USD in Niger for programmes lasting from 6 to 12 months (Margolies & Hoddinott, 2015). The activities implemented closely mirror the supply, logistics, and distribution of food commodities and supplements (AB‐CCs 1 and 2) for which we calculated monthly costs of 7.79 USD for PROCOMIDA (FFR + CSB) and 7.41 USD for *Tubaramure* (T24). A study from Chad found that the cost per beneficiary of delivering a family ration with ready‐to‐use supplementary foods for 6‐ to 36‐month‐olds was 220.40 2010 EUR per month (Puett et al., 2013). The monthly cost of PROCOMIDA's programme model that provided a full family ration with LNS was considerably less expensive at 37.60 USD. The Chad programme included a less intensive BCC component with no health systems strengthening and only served 1,700 child beneficiaries for a 5‐month period. On the basis of available evidence, we found that the monthly cost of delivering PROCOMIDA and *Tubaramure* was lower compared with other food‐assisted programmes, even though it provided a more comprehensive package of nutrition, health, and care interventions than other programmes with documented costs.

Our second objective was to compare costs and impacts of the different treatment arm–based programme models. The cost per beneficiary per percentage point reduction in stunting was 97 USD in Guatemala when the FFR was combined with CSB and 166 USD when combined with MNP. No significant effect on stunting was found in the other arms. In Burundi, reducing stunting by 1 percentage point costs 103 USD in the T24 model, 119 USD in the T18 model, and 155 USD in the TNFP model. In addition to the effects on linear growth, both programmes improved many other outcomes in children, mothers, and at the household level. Across the eight intervention arms in the two studies, the largest number of significant positive effects was found in the study arms with a significant effect on child linear growth. The Guatemala programme, however, also had undesirable effects on maternal and child anaemia and on maternal bodyweight at 24‐month post‐partum in a population without maternal underweight and a high prevalence of overweight (Heckert, Leroy, Bliznashka, Olney, & Richter, 2018). The Guatemala programme model would thus need to be adjusted before the programme is scaled up or replicated in a similar context (Leroy, Olney, & Ruel, in press). More generally, decisions on scale‐up and replication need to consider programme cost and the number and size of the programme's positive and negative effects.

Emphasizing the cost per percentage point reduction in stunting may lead to the erroneous conclusion that an investment of 100 to 150 USD per beneficiary will result in a 1 percentage point reduction in stunting that twice that investment would lead to a 2 percentage points reduction, and so on. The returns to such investments, however, are non‐linear, such that a given increase in programme spending (say, doubling) will not automatically lead to a doubling in the percentage point improvement in linear growth. This is the case for two distinct reasons. First, the proportion of fixed costs (i.e., those that do not vary with the number of beneficiaries served) relative to total costs generally decreases as the number of programme beneficiaries increases. Second, the association between inputs and biological outcomes (such as linear growth) often follows a sigmoid‐shaped curve. A low programme dose (either delivered for shorter duration, with less intensity, with fewer inputs, or with poorer quality) will not result in improved outcomes, as not all limiting nutrients and conditions have been adequately addressed; only at higher doses are observable effects found. Improving the outcome when it gets closer to its optimum level becomes more difficult and requires proportionally larger doses (and thus higher costs for each additional unit of improvement). Programme impact results from Guatemala provide evidence of this: when comparing the Guatemala programme models that varied the amount of food households received, only the ones providing the FFR had a measurable effect on linear growth (Olney et al., 2018). This impact could have been the result of the larger food ration (e.g., reduced sharing of micronutrient‐fortified food and increased availability of household resources), or the FFR may have incentivized programme participation, which may have led to greater exposure to micronutrient‐fortified food, BCC, and health‐strengthening activities. This finding is important for policy makers who are often faced with the choice between serving more people with a lighter programme or serving a smaller group of beneficiaries with a more intensive programme. Our findings suggest that below a minimum investment level (in this case, not much lower than the effective programme models), the returns drop to 0.

We intentionally did not calculate the cost per stunting case averted. Stunting is useful for population assessment and impact evaluation, but it is a poor metric of the absolute number of children affected (Leroy & Frongillo, 2019; Perumal, Bassani, & Roth, 2018). In addition to the lack of a biological or clinical basis for the arbitrary −2 *SD* cut‐off, the number of stunted children underestimates the number of children affected by an inadequate growth environment (World Health Organization Expert Committee on Physical Status, 1995). The cost per stunting case averted also assigns all programme costs to the (few) children who crossed the −2 *SD* cut‐off. Children who experienced larger HAZ improvements elsewhere in the distribution (and who may thus have incurred larger benefits) are not counted, which results in an artificial deflation of impact and an inflated cost per unit of improvement.

Our final objective was to assess what the cost savings would be if the programmes were to be implemented for a longer period. An additional 2 years of programme operation at peak enrolment would lead to a cost saving per beneficiary of approximately 11% for PROCOMIDA and 18% for *Tubaramure*. The finding that larger cost savings were found for the programme with the lower start‐up costs (17.3% in Guatemala and 10.1% in Burundi) appears paradoxical. The cost savings, however, did not only occur via the redistribution of start‐up costs over two additional years. A second mechanism is that the programme operated at peak beneficiary capacity for longer and thus delivered a more cost‐efficient programme for longer. The per beneficiary cost of food distributions during the programme roll‐out and closeout phases, for instance, was much higher than during peak enrolment as they served fewer people for only slightly lower transportation and staff costs. Interestingly, both programmes operated at peak enrolment for very short periods of time (Figure S1). In Guatemala, this was because of the time it took to initiate activities across the whole programme areas. In Burundi, this was to ensure that all beneficiaries would reach 24 months by the end of the programme.

Given the multisectoral nature of the programme and thus the multiple programme impacts, we chose to focus the analyses presented in this paper on comparing programme costs to a wide range of programme impacts. Thus, we did not conduct a full cost‐effectiveness analysis for any specific outcome (i.e., an analysis that assessed the sensitivity of the results to changes in input parameters). Important strengths of this study are the experimental design of the impact study, the prospective costing approach, and the comparison of programme costs to multiple important programme impacts.

These findings also have implications for the how investments in international assistance programmes, such as those in this study, can be modified to deliver programmes at a lower cost per beneficiary. These programmes often receive funding for fixed periods of time, during which they build a complex programme but are unable to maintain it at peak capacity for a meaningful length of time. Renewing a programme grant late in the programme cycle would not resolve this problem, as programmes need to scale‐down operations well in advance of closure. One approach could be for a 5‐year programme to be assessed after 2 years for its potential for impact before transitioning into a 7‐ or 10‐year programme. Although these conclusions are based on two multisectoral nutrition programmes, they may also apply to other international assistance programmes.

## 5 CONCLUSION

This prospective costing study of two FA‐MCHN programmes makes several contributions that further the understanding of how to optimally design and plan multisectoral nutrition programmes. To answer the question of whether to allocate a more intense programme to fewer beneficiaries (or vice versa), we found that the returns on investments in multisectoral nutrition programmes do not scale linearly and that a minimum level of investment per beneficiary is required for programmes to be effective. For example, reducing programme inputs—the size of the family ration in Guatemala and the duration of food assistance in Burundi—only marginally decreased programme activity costs (by 13–21% in Guatemala and 7–12% in Burundi), led to a lack of impact on stunting in both cases, and thus did not improve the cost per beneficiary per percentage point reduction in stunting. Further research is needed to identify the minimum investment required to achieve expected impacts on key outcomes and to better understand how differing contexts may modify this minimum investment. Further research should also explore the dynamics of programme duration and cost per beneficiary to understand how to optimize the duration of programme implementation. This study also provides insights into the cost savings of extending the duration of programme implementation. Using the ABC‐I method, we showed that lengthening the period over which the Guatemala and Burundi programmes were delivered reduced the cost per beneficiary by 11% in Guatemala and 18% in Burundi. These findings support increasing the length of investments for the delivery of effective development assistance programmes and should be considered by decision makers who wish to maximize impacts and cost‐effectiveness.

## CONFLICTS OF INTEREST

The authors declare that they have no conflicts of interest.

## CONTRIBUTIONS

JLL, DO, and MR designed and led the overall study. SR led the design of the costing study. SR and EI collected the costing data. JH, SR, and EI conducted the analysis. All authors contributed to the interpretation of the results. JH and JL drafted the manuscript and had final responsibility for submitting it. All authors read and approved the final manuscript.

## Supporting information

Table S1. Description of the nine activity‐based cost centers
Figure S1. Program enrollment patterns for the *PROCOMIDA* and *Tubaramure* programs
