# Supplementary material for: The cost of improving nutritional outcomes through food‐assisted maternal and child health and nutrition programmes in Burundi and Guatemala
Source: Matern Child Nutr. 2019 Aug 5;16(1):e12863. doi: 10.1111/mcn.12863 (PMC7038902; doi:10.1111/mcn.12863)
Supplement: Supplementary file 1 — Table S1. Description of the nine activity‐based cost centers Figure S1. Program enrollment patterns for the PROCOMIDA and Tubaramure programs [file MCN-16-e12863-s001.docx]

**Online Supplementary Table 1: Description of the nine activity-based cost centers**

| **Activity-based cost center** | **Description** |
| --- | --- |
| **Delivery of primary program components:** | |
| 1. Supply and logistics of food commodity distribution | Procurement and importing of commodities; transportation and storage of commodities from the port-of-entry to a central facility, coordination of commodity distribution, and conducting monetization of commodities to generate cash resources for the program |
| 2. Food ration and supplement distribution | Community coordination of distribution; repackaging and organization of the food rations and/or supplements; transportation of food commodities from central facilities to the distribution sites; distribution of food rations and supplements; managing and monitoring beneficiary rosters; design, procurement, and transportation of special containers or bags for distributions; testing and monitoring for allergic reactions to LNS and MNP (Guatemala only) |
| 3. Behavior change communication strategy development and execution | Formative research to develop messages; development of lesson plans, training materials, and other material; delivery of lessons to the beneficiaries; development of recipes and implementation of cooking demonstrations; implementation of the agricultural education; and implementation of microcredit groups (Burundi only) |
| 4. Institutional strengthening of health services | Trainings to improve communication between service providers and beneficiaries; technical trainings for service providers; monitoring health service activities; and administrative support to partner NGOs (Burundi only) |
| **Support, monitoring, and management activities:** | |
| 5. Monitoring and evaluation | Development of program indicators, monitoring core program components, and implementation of surveys for the evaluation |
| 6. Training and supervision of program staff | Trainings and supervisory activities for staff members in each program area |
| 7. Advocacy, promotion, and social mobilization | Obtaining the host country agreement, sensitization and approval at provincial and community levels, local ethical approval for the research, and coordination and communication with stakeholders |
| 8. Management, planning, and administration | Financial and administrative support that include human resources support, procurement, proposal writing and coordinating with local offices, office management, coordination among the different NGO offices, and finance and administrative support to partner NGOs (Burundi only) |
| 9. Systematic information management (Guatemala only) | Development and implementing the information systems to track commodities, manage beneficiary information, and, monitor program coverage |

Note: Descriptions apply to both programs unless otherwise noted. The cost of food rations and supplements are not included in these activities.

**Online Supplementary Figure 1 Program enrollment patterns for the *PROCOMIDA* and *Tubaramure* programs**

Note: Values are based on monitoring data provided by the programs and supplemented by the authors’ estimates for time periods with incomplete information. At the start of the program, T*ubaramure* enrolled only households with a pregnant mother or a child younger than 6 months, and enrollment ended when beneficiaries would no longer reach 24 months before the program end. It delivered the program across the full delivery area almost immediately (with the exception of the areas selected for the impact evaluation). At the start of PROCOMIDA, however, initial enrollment was open to households with a pregnant woman or child younger than 24 months old, and enrollment ended one year before the end of the program. It also took approximately 6 months to initiate activities across the whole program areas.
